# Interleukin 1 family dysregulation in serum and peritoneal fluid of ovarian cancer patients – potential clinical implications

**DOI:** 10.3389/fonc.2025.1653017

**Published:** 2026-01-05

**Authors:** Sebastian Stępień, Marta Smycz-Kubańska, Maria-Laura Morawiec, Kamil Radosław Seweryn, Aleksandra Mielczarek-Palacz

**Affiliations:** Department of Immunology and Serology, Faculty of Pharmaceutical Sciences in Sosnowiec, Medical University of Silesia in Katowice, Katowice, Poland

**Keywords:** cytokines, gynecological cancer, IL-1, inflammation, interleukins, ovarian cancer

## Abstract

In the pathomechanism of ovarian cancer development, an important role is attributed to the chronic inflammatory process. Still, despite numerous studies, it has not been fully elucidated how immune inflammatory processes influence the development of ovarian cancer. Immune system mediators – cytokines, especially interleukin-1 family members – are involved in interactions between cancer cells and immune cells. Therefore, learning about the relationship between the coexistence of inflammatory and neoplastic processes in ovarian cancer involving these molecules may contribute to a better understanding of the importance of the studied parameters in the pathogenesis of cancer, which may also translate into improved clinical efficacy. In the present study, for the first time, the analysis between the levels of all 11 members of the IL-1 family (IL-1α, IL-1β, IL-18, IL-33, IL-36α, IL-36β, IL-36γ, IL-37, IL-1Ra, IL-36Ra, IL-38) has been made, which belong to the main inflammatory cytokines, in both serum and peritoneal fluid of ovarian cancer patients. The analysis revealed differences in the concentration of individual members of IL-1 in both serum and peritoneal fluid between the study group and the reference group, as well as between G1, G2 and G3 stages of ovarian cancer, and differences in the ratio between the interleukins studied. The results indicate the involvement of the studied parameters in the pathomechanism of ovarian cancer development and the regulation of stimulation of inflammation accompanying ovarian cancer. Disruption of the secretion of secreted cytokines in serum and peritoneal fluid in patients with ovarian cancer may lead to their pro-inflammatory activation, indicating that modulation of cytokines of the IL-1 family may affect the course of the inflammatory process that accompanies the development of cancer. The observed perturbations involving the cytokines studied may represent one of the more important immune mechanisms that promote the development of this cancer, indicating the need for new complex therapies targeting a wide variety of immune response mechanisms, but this requires further, more advanced research.

## Graphical Abstract

**Figure:**
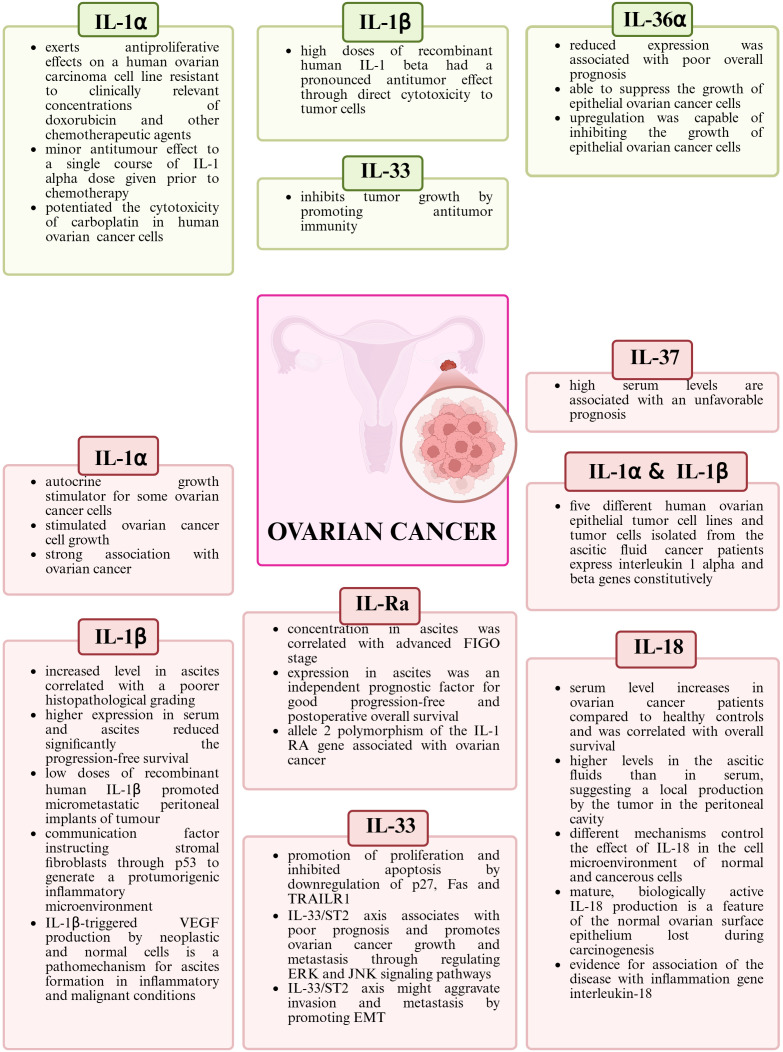
The role of IL-1 family members in the pathogenesis of ovarian cancer. Green color indicates anti-tumor activity, while red color indicates pro-tumor activity. Created in https://BioRender.com.

## 1 Introduction

Ovarian cancer (OC) among gynecological cancers is characterized by a high morbidity rate (number of new cases in 2020 vs. number of new cases in 2022 according to GLOBOCAN; 313,959 vs. 324,398), a high mortality rate (number of cases in 2020 vs. number of cases in 2022 according to GLOBOCAN; 207,252 vs. 206,839) (1, 2), diagnosis of patients at a late stage of ovarian cancer (>70% of cases are diagnosed at FIGO (International Federation of Gynecology and Obstetrics) stage III or IV) (3, 4), high recurrence rate (up to 70% of patients within the first three years) (3), low overall survival rate (five-year overall survival rate <50%) (5), resulting in ovarian cancer being ranked fifth in terms of cancer mortality rate among women worldwide (6). Furthermore, it is estimated that ovarian cancer mortality will continue to rise until 2040 (4).

Despite many years of extensive research, the role of the immune system in the pathogenesis of ovarian cancer remains unclear. Recent studies on the biology of this cancer have shown that interactions between immune system cells and cancer cells are important in their formation and development. These interactions include both direct interactions between cells and indirect interactions via secreted soluble mediators of the immune system, including cytokines (7–9).

Chronic inflammation is considered to play an important role in the pathogenesis of ovarian cancer. It has been shown that inflammation is associated with poor prognosis and shorter survival in patients with OC (10–12). In OC, inflammation is thought to arise with the involvement of reactive oxygen species (ROS), cytokines, chemokines and growth factors. These molecules are synthesized by the ovaries, immune cells and tumor cells. The resulting inflammatory microenvironment can damage DNA, initiate pro-inflammatory and pro-tumor signaling pathways, induce epigenetic modifications, chromosomal mutations and interfere with the immune response directed against tumor cells (12).

It is now well acknowledged that inflammation is a leading factor in the development and progression of tumors at many stages, particularly during proliferation, invasion, angiogenesis and metastasis. Furthermore, the cytokines released associated with coexistent inflammation, which are up-regulated by many cells, including those in the tumor microenvironment, are capable of directly affecting tumor cells, thereby exacerbating the various stages of carcinogenesis (13–18).

**Figure 1:**
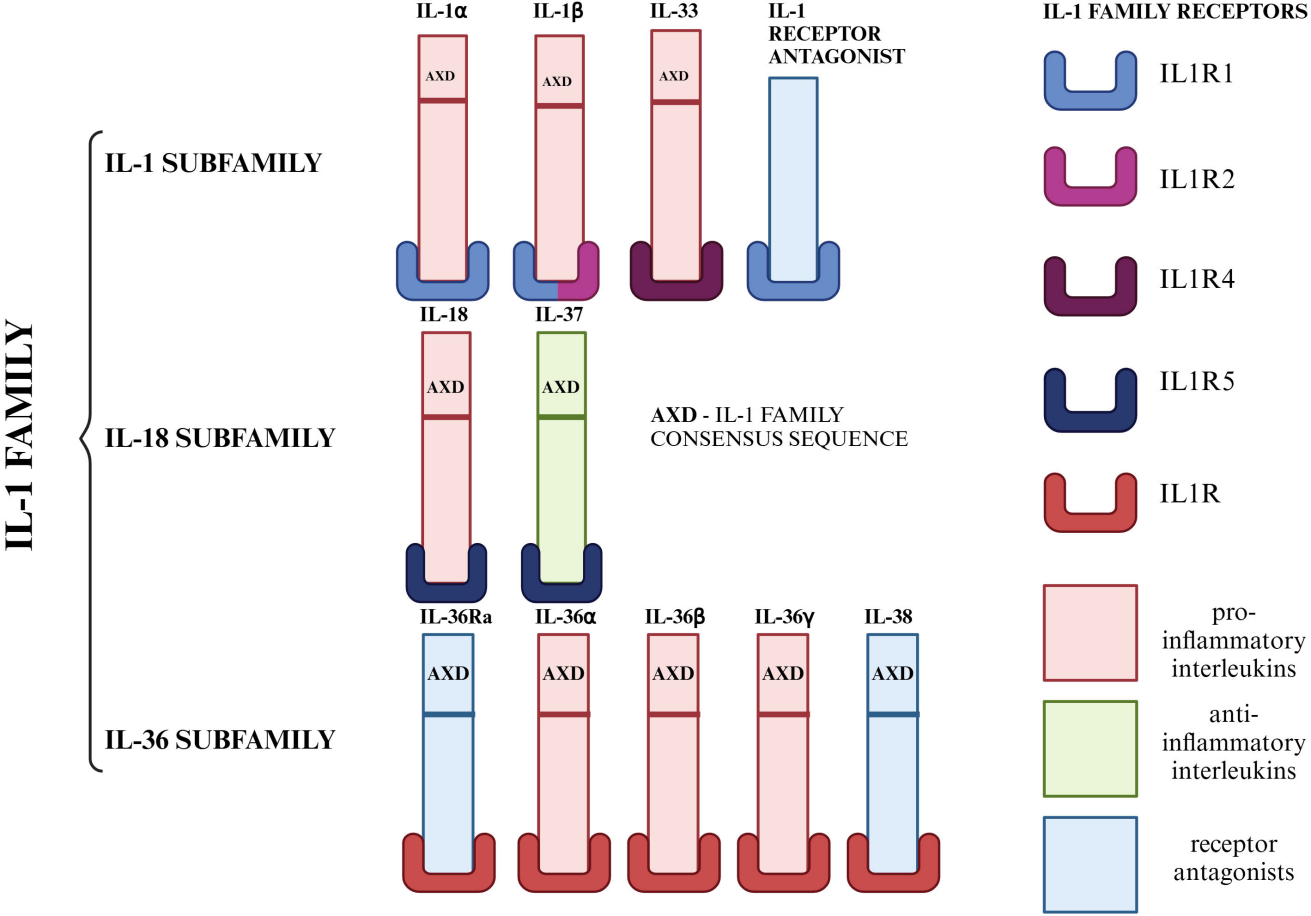
Division of IL-1 family members into subfamilies. Due to the AXD consensus sequence, we can divide the IL-1 family into 3 subfamilies: the IL-1 subfamily (IL-1α, IL-1β, IL-33), the IL-18 subfamily (IL-18, IL-37), the IL-36 subfamily (IL-36α, IL-36β, IL-36γ) and IL-1Ra, which was not included in any of the subfamilies due to the lack of AXD space. On the other hand, the IL-1 family can be divided according to the role it plays in the inflammatory process. Taking this criterion into account, we distinguish seven pro-inflammatory molecules (IL-1α, IL-1β, IL-18, IL-33, IL-36α, IL-36β, IL-36γ), one anti-inflammatory molecule (IL-37) and three molecules with antagonistic properties (IL-1Ra, IL-36Ra, IL-38) (19–27). Created in https://BioRender.com.

Members of interleukin 1 (IL-1) play an important role in the regulation of the inflammatory process. The IL-1 family includes 11 molecules, characterized by a similar structure and mechanism of interaction with specific receptors. The cytokines of this family are produced as inactive cytoplasmic precursors, containing an AXD (IL-1 family consensus sequence). Based on the AXD consensus sequence, we can divide the IL-1 family into 3 subfamilies: the IL-1 subfamily, the IL-18 subfamily and the IL-36 subfamily (19–27). Among this family of interleukins, we can also distinguish pro-inflammatory (IL-1α, IL-1β, IL-18, IL-33, IL-36α, IL-36β, IL-36γ), anti-inflammatory (IL-37) and cytokines with antagonistic properties (IL-1Ra, IL-36Ra, IL-38) (19, 20, 22). This subdivision, which also presents the specific receptors for each member of the IL-1 family mediated by the biological function of these cytokines in the body, is shown in **Figure 1**.

**Figure 2:**
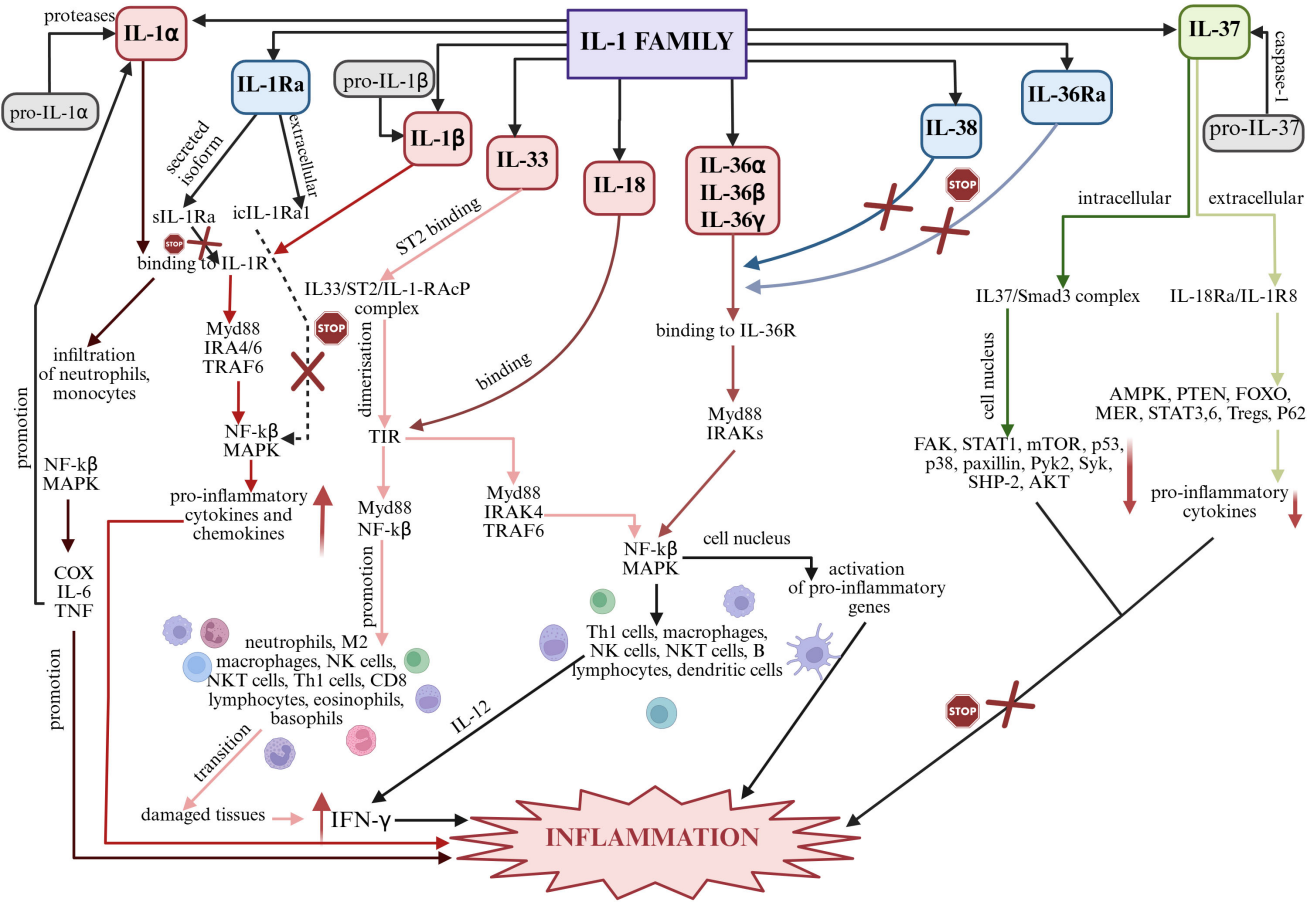
Summary of signaling pathways of individual IL-1 family members in inflammation. Created in https://BioRender.com. AKT – protein kinase B; AMPK – AMP-activated protein kinase; COX – cyclooxygenase type-2; FAK – focal adhesion kinase; FOXO – forkhead box O; IFN-γ – interferon gamma; IL-6 – interleukin 6; IL-12 – interleukin 12; IRAK – interleukin receptor-associated kinase; MAPK – mitogen-activated protein kinases; MER – myeloid-epithelial-reproductive tyrosine kinase; mTOR – mechanistic target of rapamycin; Myd88 – myeloid differentiation primary response 88; NF-kβ – nuclear factor kβ; p38 – p38 mitogen-activated protein kinases; p53 – tumor protein p53; P62 – nuclear pore glycoprotein p62; Pyk2 – protein tyrosine kinase 2 beta; SHP-2 – K-box region and MADS-box transcription factor family protein; STAT 1, 3 i 6 – signal transducer and activator of transcription 1, 3 i 6; Syk – spleen associated tyrosine kinase; TIR – Toll-interleukin receptor; TNF – tumor necrosis factor; TRAF6 - TNF receptor associated factor 6. **IL-1α:** Under the influence of proteases, pro-IL-1α is converted into bioactive IL-1α, which, via IL-1R, induces inflammatory responses, affecting neutrophils and monocytes in particular. Subsequently, due to the induction of NF-kβ and MAPK, the inflammatory mediators COX-2, IL-6, TNF are produced, which promote the development of inflammation and further promote the production of IL-1α and IL-1β, enhancing pro-inflammatory processes (24, 31). IL-1β: Biologically inactive pro-IL-1β is converted to bioactive IL-1β and subsequently binds to IL-1R. Myd88-, IRAK4-, IRAK6-, TRAF6-mediated signaling is stimulated and the NF-kβ and MAPK pathway is activated, affecting the up-regulation of inflammatory genes (31). IL-33: IL-33 binds to the specific ST2 receptor and a complex is formed that causes dimerization of TIR. The resulting complex stimulates intracellular signaling through Myd88, IRAK1 and IRAK4 and TRAF6. MAP and NF-kβ are then activated, which stimulate the inflammatory cascade (32, 33). IL-18: The inactive pro-IL-18 precursor specifically under the influence of caspase-1 and NLRP3 is converted into biologically active IL-18, which interacts via IL-18R, containing a TIR domain. Subsequently, under the influence of Myd88, IRAK1, IRAK6, TRAF6 are recruited and NF-kβ and the MAPK cascade are activated, which stimulate cells of the immune system. IL-12-mediated synthesis of IFN-γ occurs, leading to the development of the inflammatory process (34). IL-36α, IL-36β, IL-36γ: Biologically inactive interleukin precursors, under the influence of caspase-1 or specifically by cathepsin G, elastase and proteinase-3, become active molecules that bind to the receptor to form a complex. Myd88 and IRAKs then interact with the complex causing activation of NF-kβ and MAPKs. NF-kβ in the cell nucleus activates pro-inflammatory genes (31). IL-38 and IL-36Ra: The biological function of these interleukins includes blocking the binding of IL-36 cytokines (IL-36α, IL-36β and IL-36γ) to IL-36R. Due to their antagonistic properties, these interleukins may exhibit anti-inflammatory effects (27). IL-37: INTRACELLULAR – The inactive pro-IL-37 precursor under the influence of caspase-1 is converted to biologically active IL-37, which forms a complex with Smad3. Down-regulation of individual inflammatory pathways then occurs, preventing the development of inflammation; EXTRACELLULAR – The inactive pro-IL-37 precursor under the influence of caspase-1 is converted into biologically active IL-37, which binds to IL-18Rα and a complex is formed that prevents the activation of inflammatory pathways and thus the development of inflammation (31, 33). IL-1Ra: As a secreted form, sIL-1Ra competitively inhibits the binding of IL-1α and IL-1β to the IL-1R1 receptor, thereby inhibiting IL-1-mediated signaling. As an intracellular form, icIL-1Ra1’s signaling mechanism is not entirely clear. It is thought to inhibit NF-kβ activation, by blocking IL-1 binding to IL-1R1 (35).

The biological role of IL-1 family interleukins is regulated at the level of their synthesis, the existence of IL-1 family mediators, specific IL-1 receptors (IL-1R) and inflammatory cytokines. Signaling is dependent on factors associated with the inflammatory response, particularly involving nuclear factor kβ (NF-kβ), mitogen-associated protein kinase (MAPK), protein kinase B (AKT), and interleukin receptor-associated kinase (IRAK) (28–30). Signaling pathways in the inflammatory process in which individual members of the IL-1 family play a key role are shown in **Figure 2**.

Members of the IL-1 family exhibit pleiotropic effects in the organism; they are mediators of inflammation in the body (22, 24, 36, 37, 54), they are involved in the innate and acquired immune response (22), they influence the regulation of angiogenesis and vascular permeability (23, 36), and their action has been described as a pathological mediator of many diseases, especially those with autoimmune, infectious and degenerative backgrounds (36), as well as cancer (23, 36). Moreover, they also provide an important function in the regeneration of damaged tissues and the maintenance of systemic homeostasis (26, 37). Moreover, members of the IL-1 family exhibit synergistic as well as antagonistic effects (24).

In the case of the carcinogenesis process of many cancers, IL-1 members exhibit dualistic properties, showing both anti-tumor and pro-tumor effects (29, 31, 38). Baker et al. (31) indicate that the importance of individual IL-1 members varies and depends on the tissue and organ affected by the process of carcinogenesis, the state of inflammation, the stage of cancer development, and the type of cells synthesizing these cytokines.

Therefore, it seems necessary to conduct further research on OC biology in order to better understand and elucidate the role of inflammation in OC pathogenesis, with the potential to contribute to the development of new diagnostic and therapeutic paradigms, thereby improving treatment efficiency and survival of ovarian cancer patients. Therefore, the aim of the present study was to analyze the concentrations of all 11 members of the IL-1 family (IL-1α, IL-1β, IL-18, IL-33, IL-36α, IL-36β, IL-36γ, IL-37, IL-1Ra, IL-36Ra, IL-38) in the serum and peritoneal fluid of patients with ovarian cancer and to determine the relationship between the parameters studied and the degree of histological differentiation of ovarian cancer.

## 2 Materials and methods

### 2.1 Study population

Thirty-two patients aged 48 to 89 years (mean age ± SD, 67.72 ± 12.50 years) with clinically diagnosed and histopathologically confirmed serous ovarian cancer (*Cystadenocarcinoma papillare serosum*) stage IIIC according to the FIGO classification were included in the study group. The inclusion criteria included newly diagnosed disease confirmed by histopathological examination, age over 18 years, no drug treatment within the last 3 months of specimen collection and an informed consent to participate in the study. Exclusion criteria included the coexistence of other conditions of the reproductive organs and other chronic diseases and lack of consent to participate in the study. The diagnosis of ovarian cancer was made on the basis of clinical symptoms, gynecological examination findings, laboratory tests and histopathological examination. The criteria recommended by FIGO were used to assess the stage. The grade of histological differentiation of the cancer (grading) was presented according to the following scale:

- G1 – highly differentiated,
- G2 – moderately differentiated,
- G3 – poorly differentiated.

Considering the above criterion, the participation of women in the study group is as follows: G1–11 patients (34.4%), G2–10 patients (31.2%), G3–11 patients (34.4%). All the patients studied were hospitalized in the Gynecology and Obstetrics Department with Pregnancy Pathology and Gynecology Oncology Subdivisions, Provincial Specialist Hospital Blessed Virgin Mary (Częstochowa, Poland). The material collected from the patients was venous blood and peritoneal fluid.

### 2.2 Reference population

The reference group in this study consisted of 18 female patients aged 19 to 77 years (mean age ± SD, 48.78 ± 12.2 years). These patients were diagnosed with a benign ovarian tumor (*Cystadenoma serosum*). Inclusion criteria were age over 18 years, general health as good, no drug treatment within the last 3 months of material collection and an informed consent to participate in the study. Exclusion criteria included coexisting chronic diseases, especially cancer, and lack of consent to participate in the study. The material collected from the patients was venous blood.

### 2.3 Serum preparation

Blood was collected from women after clinical diagnosis was established and before surgery. Blood was collected in the morning from the elbow vein into a tube containing a clotting activator to obtain serum. 30 minutes after collection, the tubes containing the material were centrifuged at 1500 x g for 15 minutes at room temperature. The resulting serum was separated from the clot, frozen at -80°C and stored until analysis.

### 2.4 Peritoneal fluid preparation

Peritoneal fluid was collected during laparoscopy for bacteriological examination. The collected material was centrifuged at 1500xg for 10 minutes at 4°C. The resulting supernatant was pooled, frozen at -80°C and stored until analysis.

### 2.5 ELISA tests

IL-1α, IL-1β and IL-33 concentrations were determined using sandwich ELISAs with the Diaclone Human ELISA Kit from Diaclone SAS (Besançon, France), while IL-18, IL-37, IL-36α, IL-36β, IL-36γ, IL-38, IL-36Ra and IL-1Ra concentrations were determined using sandwich ELISAs with the CLOUD-CLONE ELISA Kit from Cloud-Clone Corp. (Houston, USA). All assays were performed according to the manufacturer’s attached protocols. The characteristics of the individual assays used in this study are shown in **Table 1**.

**Table 1:** Laboratory characteristics of the tests used for the assays.

Parameters	Test name	Unit	Analytical sensitivity	Detection range	Precision	
					Intra-assay	Inter-assay
IL-1α	Human IL-1α ELISA Kit manufactured by Diaclone SAS (Cat. No. 850.005.096)	pg/ml	10	31.25-1000	CV<4.3%	CV<7.3%
IL-1β	Human IL-1β ELISA Kit manufactured by Diaclone SAS (Cat. No. 850.006.096)	pg/ml	6.5	15.625-500	CV<10.5%	CV<11.7%
IL-33	Human IL-33 ELISA Kit manufactured by Diaclone SAS (Cat. No. 873.020.096)	pg/ml	12.2	31.25-1000	CV<7.57%	CV<15.0%
IL-18	Enzyme-linked Immunosorbent Assay Kit for Interleukin 18 manufactured by Cloud-Clone Corp. (Cat. No. SEA 064Hu)	pg/ml	5.9	15.9-1000	CV<10%	CV<12%
IL-37	Enzyme-linked Immunosorbent Assay Kit for Interleukin 1 Zeta (IL1z) manufactured by Cloud-Clone Corp. (Cat. No. SEE842Hu)	pg/ml	3.2	7.8-500	CV<10%	CV<12%
IL-36α	Enzyme-linked Immunosorbent Assay Kit for Interleukin 1 Epsilon (IL1e) manufactured by Cloud-Clone Corp. (Cat. No. SEE843Hu)	pg/ml	6.3	15.6-1000	CV<10%	CV<12%
IL-36β	Enzyme-linked Immunosorbent Assay Kit for Interleukin 1 Eta (IL1h) manufactured by Cloud-Clone Corp. (Cat. No. SED134Hu)	pg/ml	3.1	7.8-500	CV<10%	CV<12%
IL-36γ	Enzyme-linked Immunosorbent Assay Kit for Interleukin 1 Family, Member 9 (IL1F9) manufactured by Cloud-Clone Corp. (Cat. No. SEL621Hu)	pg/ml	6.5	15.6-1000	CV<10%	CV<12%
IL-38	Enzyme-linked Immunosorbent Assay Kit for Interleukin 1 Theta (IL1q) manufactured by Cloud-Clone Corp. (Cat. No. SEQ458Hu)	pg/ml	3.2	7.8-500	CV<10%	CV<12%
IL-36Ra	Enzyme-linked Immunosorbent Assay Kit for Interleukin 1 Delta (FIL1d) manufactured by Cloud-Clone Corp. (Cat. No. SEA566Hu)	pg/ml	6.1	15.6-1000	CV<10%	CV<12%
IL-1Ra	Enzyme-linked Immunosorbent Assay Kit for Interleukin 1 Receptor Antagonist (IL1RA) manufactured by Cloud-Clone Corp. (Cat. No. SEA223Hu)	pg/ml	12.1	31.2-2000	CV<10%	CV<12%

The following data are from protocols provided by the test manufacturers.

### 2.6 Statistical analysis

The results obtained were subjected to statistical analysis using Statistica 13.3 software (StatSoft Polska Sp. z o.o.). Basic statistical parameters were determined for the parameters studied, including: mean, median, standard deviation, minimum value, maximum value, and interquartile range. The normality of the distribution of the studied variables was checked using the Shapiro-Wilk test. In the case of normally distributed data, Student’s t-test was used, while non-normally distributed data were analyzed with the Mann–Whitney U test.

Correlations were tested using Spearman’s rank correlation test and presented as correlation coefficient (r). The level of statistical significance was taken as p<0,05. Correlations were classified as negative or positive, and the correlation index (r) was used to define correlations as faint (r<0,1), weak (ranging from 0,1 to 0,3), moderate (ranging from 0,3 to 0,5), strong (ranging from 0,5 to 0,7) or very strong (r>0,7).

The ratio between interleukins was obtained using the ANOVA test. Due to the normal distribution of the analyzed interleukin ratios, parametic ANOVA was used. When p<0.05, a *post-hoc* test was performed to determine between which analyzed groups there was a statistically significant difference. The results are presented as a box-plot graph.

The study was conducted according to the guidelines of the Declaration of Helsinki, and approved by the Ethics Committee of Medical University of Silesia in Katowice, Poland (protocol code KNW/0022/KB1/49/19). All patients agreed to participate in the present study and provided written informed consent.

## 3 Results

Analyses were performed on the concentration of all members of the IL-1 family (IL-1α, IL-1β, IL-33, IL-18, IL-37, IL-36α, IL-36β, IL-36γ, IL-38, IL-36Ra and IL-1Ra) in the serum and peritoneal fluid of patients with ovarian cancer (representing the study group) and patients with benign ovarian tumors (representing the reference group). The data obtained from the measurements is presented in **Table 2**.

**Table 2:** Basic descriptive statistics of the analyzed parameters.

Parameters	Group	Analyzed material	Statistical parameters							
			N	m	s	Me	Q_1_	Q_3_	x_min_	x_max_
IL-1α (pg/ml)	Study group	serum	32	31.68	22.92	23.82	19.76	28.37	13.65	97.90
		peritoneal fluid	32	26.04	12.17	21.69	18.01	28.80	14.08	64.66
	Reference group	serum	16	17.77	3.11	18.14	16.58	19.73	10.03	21.82
IL-1β (pg/ml)	Study group	serum	32	18.33	5.34	16.14	15.53	17.51	14.93	31.98
		peritoneal fluid	32	19.19	6.10	16.75	15.38	20.10	14.32	43.25
	Reference group	serum	16	15.67	0.94	15.53	14.93	16.14	14.62	17.97
IL-33 (pg/ml)	Study group	serum	32	142.97	102.68	120.24	89.40	141.42	15.52	473.28
		peritoneal fluid	32	72.92	32.43	71.05	43.37	94.92	17.28	136.15
	Reference group	serum	16	15.27	2.88	14.82	12.81	17.74	12.36	19.35
IL-18 (pg/ml)	Study group	serum	32	96.92	97.81	52.48	12.30	166.06	6.37	287.25
		peritoneal fluid	32	37.73	38.27	17.88	9.52	70.78	6.25	154.00
	Reference group	serum	16	8.22	1.26	8.21	7.50	9.35	6.14	9.80
IL-37 (pg/ml)	Study group	serum	32	78.09	59.30	62.78	35.90	108.21	10.12	236.14
		peritoneal fluid	32	43.56	10.05	41.75	36.26	48.38	28.33	70.98
	Reference group	serum	16	13.26	4.17	13.89	9.34	16.59	6.73	19.56
IL-36α (pg/ml)	Study group	serum	32	22.75	20.50	15.26	7.15	28.78	6.40	88.20
		peritoneal fluid	32	14.83	11.98	9.64	7.40	20.80	6.60	68.21
	Reference group	serum	16	7.52	0.84	7.35	6.98	7.77	6.55	9.78
IL-36β (pg/ml)	Study group	serum	32	6.79	0.50	6.72	6.72	6.88	5.00	8.44
		peritoneal fluid	32	6.86	0.89	6.72	6.56	7.03	5.00	9.22
	Reference group	serum	16	6.56	0.28	6.56	6.56	6.72	5.63	6.88
IL-36γ (pg/ml)	Study group	serum	32	149.02	107.64	140.15	62.48	230.22	24.50	360.87
		peritoneal fluid	32	471.82	53.62	270.06	90.17	575.39	5.29	1946.15
	Reference group	serum	16	66.94	36.54	65.63	40.69	82.38	8.80	131.66
IL-38 (pg/ml)	Study group	serum	32	7.90	0.98	7.50	7.33	7.83	7.17	10.71
		peritoneal fluid	32	7.44	0.32	7.42	7.17	7.67	6.83	8.17
	Reference group	serum	16	6.54	1.11	7.23	5.33	7.42	5.07	7.83
IL-36Ra (pg/ml)	Study group	serum	32	17.24	3.28	17.31	14.62	19.23	12.31	24.62
		peritoneal fluid	32	16.86	2.83	17.50	13.85	18.65	12.69	22.31
	Reference group	serum	16	19.33	1.67	18.65	18.08	20.19	17.69	22.31
IL-1Ra (pg/ml)	Study group	serum	32	110.53	41.99	104.11	85.03	129.90	14.68	219.00
		peritoneal fluid	32	104.51	39.10	98.23	74.80	125.47	46.46	222.52
	Reference group	serum	16	63.02	15.98	59.46	49.50	75.08	40.18	91.46

N - abundance, m - mean, s - standard deviation, Me - median, Q1 - lower quartile, Q3 - upper quartile, xmin - minimum value, xmax - maximum value.

IL-1 family member concentrations in serum and peritoneal fluid of women with ovarian cancer were assessed according to histological differentiation stage (FIGO).

### 3.1 Analysis of IL-1 family member concentrations in serum and peritoneal fluid

**Figure 3:**
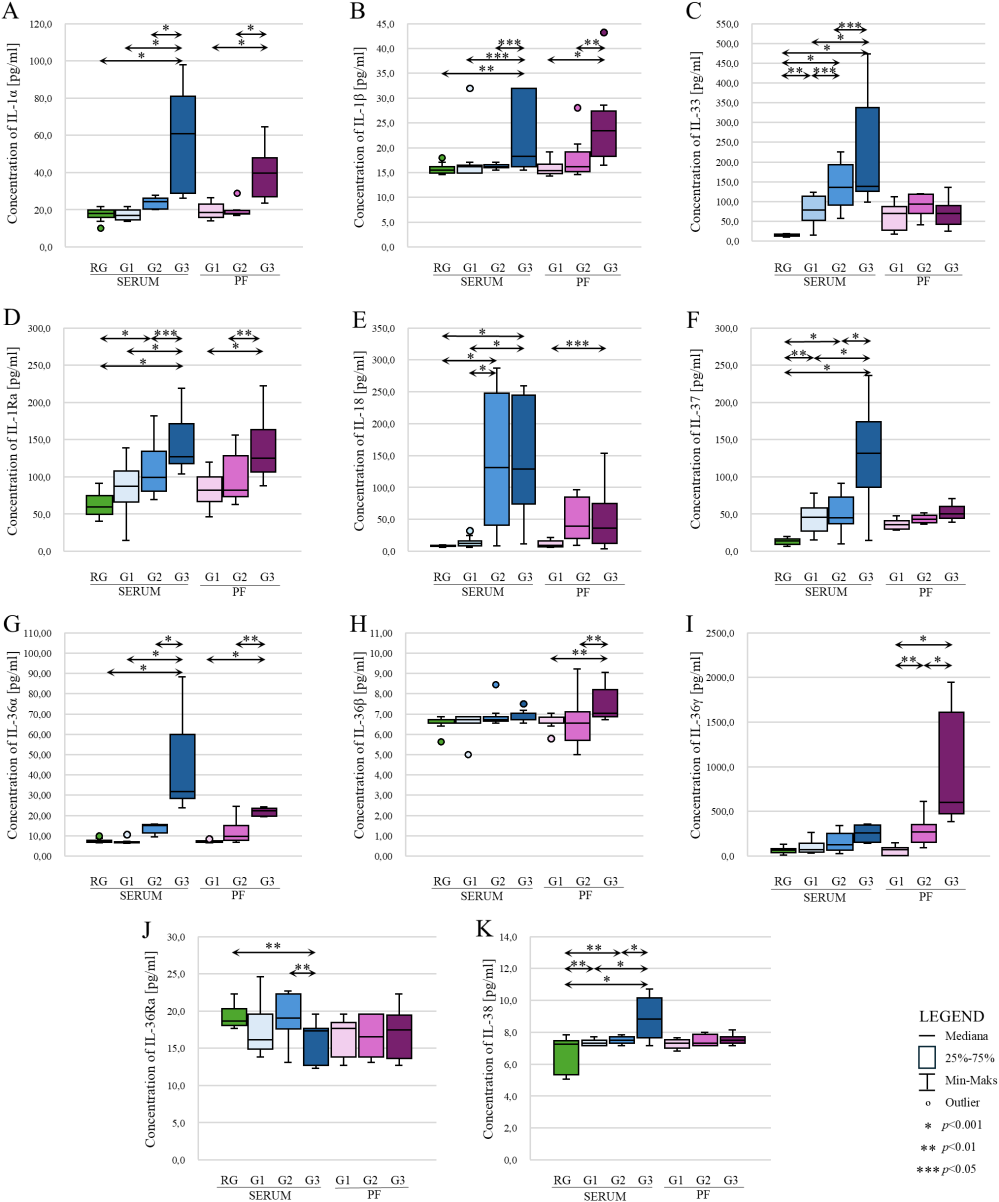
Comparison of concentrations of individual IL-1 family members: IL-1α **(A)**, IL-1β **(B)**, IL-33 **(C)**, IL-1Ra **(D)**, IL-18 **(E)**, IL-37 **(F)**, IL-36α **(G)**, IL-36β **(H)**, IL-36γ **(I)**, IL-36Ra **(J)** and IL-38 **(K)** in serum and peritoneal fluid in patients with benign ovarian tumor (reference group) and patients with ovarian cancer (study group) according to histological differentiation stage (FIGO). In the figure, only data with statistical significance are indicated by arrows. RG - reference group, PF - peritoneal fluid, G1 - highly differentiated, G2 - moderately differentiated, G3 - poorly differentiated, * - p<0.001, ** - p<0.01, *** - p<0.05.

Statistical analysis showed statistically significantly higher IL-1α concentrations in serum of patients with ovarian cancer stage G3 compared to patients in the reference group (p<0.001) and patients in the study group stage G1 vs. G3 (p<0.001) and G2 vs. G3 (p<0.001). Serum IL-1α levels were not significantly different between patients in the reference group vs. G1, reference group vs. G2 and G1vs. G2 patients (p>0.05). The peritoneal fluid showed statistically significantly higher concentrations of the analyzed parameter in patients with ovarian cancer grade G3 compared to patients with G1 and G2 (p<0.001). There was no statistically significant difference in the concentration of IL-1α in the peritoneal fluid in G1 vs. G2 patients (p>0.05). The results obtained are shown in **Figure 3A**.

A statistically significant higher concentration of IL-1β in the serum of patients with stage G3 ovarian cancer was demonstrated compared to the reference group (p<0.01), patients with stage G1 and G2 ovarian cancer (both p<0.05). Assessing IL-1β levels in the peritoneal fluid of women also showed, similar to IL-1α, statistically significant higher levels of IL-1β in the peritoneal fluid of women with ovarian cancer stage G3 vs. G1 (p<0.001) and G3 vs. G2. (p<0,01). There was no statistically significant difference in the concentration of this interleukin between G1 and G2 stages (p>0.05). The results obtained are shown in **Figure 3B**.

The analysis of IL-33 serum levels in patients showed a statistically significant higher IL-33 concentration in patients in grades G1, G2 and G3 compared to the control group, p value was p<0.01, p<0.001 and p<0.001, respectively. In addition, the analysis showed a statistically significant difference in concentration between grades (G1 vs. G2; p<0.05, G1 vs. G3; p<0.001; G2 vs. G3, p<0.05). In contrast, analysis showed no statistically significant differences in peritoneal fluid IL-33 levels between histological differentiation grades of ovarian cancer. In each case, p>0.05. The results obtained are shown in **Figure 3C**.

Evaluation of IL-1Ra levels showed statistically significant higher levels in patients with ovarian cancer stage G3 compared to the reference group (p<0.001), G3 vs. G1 (p<0.001), G3 vs. G2 (p<0.05). Higher concentrations were also shown in G2 vs. G1 (p<0.001). There was no significant change in IL-1Ra concentration between G1 and the reference group (p>0.05). In contrast, examination of IL-1Ra levels in the peritoneal fluid of patients diagnosed with ovarian cancer showed statistically significantly higher IL-1β levels among women in stage G3 vs. G1 (p<0.001) and G3 vs. G2 (p<0.01). There was no statistically significant difference in the concentration of the tested interleukin between G1 and G2 (p>0.05). The results obtained are shown in **Figure 3D**.

There was a statistically significant higher serum IL-18 concentration in G2 and G3 patients compared to the reference group and compared to G1 patients. In all cases, the p value was p<0.001. No significant difference was found between G2 and G3 (p>0.05). The peritoneal fluid only showed statistically significantly higher concentrations in stage G3 compared to stage G1 (p<0.05). The results obtained are shown in **Figure 3E**.

In the analysis of serum IL-37 concentrations, there was a statistically significant difference between the reference group and G1 (p<0.01), G2 (p<0.001), G3 (p<0.001). Serum concentrations were also shown to be significantly higher between G1 vs. G2, G1 vs. G3 and G2 vs. G3 - in all cases mentioned, statistical significance was p<0.001. Analyzing the concentration of IL-37 in the peritoneal fluid showed no statistically significant differences between G1, G2 and G3 grades (p>0.05), although the box-plot shows a change in the concentration of this interleukin with increasing histological differentiation of the ovarian cancer, but, as mentioned earlier, this change is not statistically significant. The results obtained are shown in **Figure 3F**.

Statistical evaluation of serum IL-36α levels showed statistically significantly higher IL-36α levels in patients at stage G3 compared to the reference group (p<0.001), relative to stage G1 (p<0.001) and to stage G2 (p<0.001). There was no significant difference in concentration in RG vs. G1, RG vs. G2 and G1 vs. G2 (p>0.05). When analyzing IL-36α concentrations in the peritoneal fluid, statistical analysis only showed significantly higher concentrations in grade G3 compared to grade G1 (p<0.001) and to grade G2 (p<0.01). The results obtained are shown in **Figure 3G**.

Statistical analysis of IL-36β serum levels in patients in the reference group and the study group showed no statistically significant difference (p>0.05). Moreover, IL-36β concentrations were low in each group (reference group (m) vs. study group (m), 6.56 vs. 6.79 pg/ml). A statistically significant difference in concentration was only seen in the peritoneal fluid between grade G1 vs. G2 and G1 vs. G3. Statistical significance in both cases was p<0.01. The results obtained are shown in **Figure 3H**.

In the analysis of IL-36γ serum levels in patients with benign ovarian tumor (reference group) vs. patients with ovarian cancer (study group), an increase in IL-36γ levels was noted in the ovarian cancer group. Furthermore, an increase in concentration was also noted between the different histological differentiation grades of ovarian cancer. Unfortunately, statistical analysis showed that the increase in serum concentrations of this interleukin was not statistically significant in each case (p>0.05). A statistically significant difference in the concentration of the tested interleukin was shown in the peritoneal fluid. A statistically significant higher concentration was observed in grade G2 to G1 (p<0.01), G3 to G1 (p<0.001) and G3 to G2 (p<00.001). The results obtained are shown in **Figure 3I**.

Analysis of serum IL-36Ra concentrations in the reference group compared to the study group showed only statistically significantly lower concentrations in the study group at grade G3 (RG vs. G3; p<0.01). There was no difference in the concentration of the analyzed parameter between the reference group and the study group in grades G1 and G2 (p>0.05). Evaluation of the concentration of the interleukin analyzed in the peritoneal fluid showed no statistical significance between grades G1, G2 and G3. In each case, p>0.05. The results obtained are shown in **Figure 3J**.

A statistically significant higher concentration of IL-38 was evidenced among the group of patients in grade G3 compared to the reference group, to the group of patients in grades G1 and G2. In the present cases, the p value was p<0.001. In addition, a statistically significant difference was shown in concentrations in G1 and G2 stages compared to the control group (p<0.01). For the analysis of IL-38 concentration in the peritoneal fluid, no statistically significant difference in concentration was shown between grades G1, G2 and G3. In each case, the p value was p>0.05. The results obtained are shown in **Figure 3K**.

### 3.2 Correlation analysis of serum concentrations of IL-1α, IL-1β, IL-33, IL-18, IL-37, IL-36α, IL-36β, IL-36γ, IL-38, IL-36Ra and IL-1Ra in relation to the concentrations of these parameters in the peritoneal fluid among the study group

**Figure 4:**
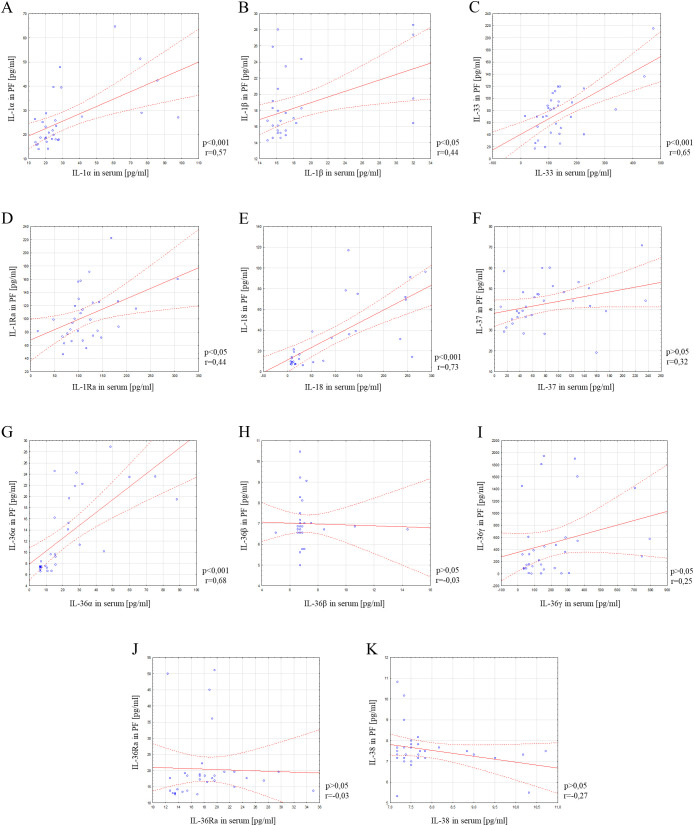
Correlation analysis showing the association between serum and peritoneal fluid IL-1α **(A)**, IL-1β **(B)**, IL-33 **(C)**, IL-1Ra **(D)**, IL-18 **(E)**, IL-37 **(F)**, IL-36α **(G)**, IL-36β **(H)**, IL-36γ **(I)**, IL-36Ra **(J)** and IL-38 **(K)** concentrations. Solid line - regression line, dotted line - 95% confidence interval, sign ° - values noted during the analysis, p - level of statistical significance, r - correlation coefficient, PF - peritoneal fluid.

In the next part of the study, correlation analysis of the studied parameters was performed using Spearman’s rank correlation test. Positive statistically significant correlations were found between serum and peritoneal fluid concentrations of ovarian cancer patients in the case of IL-1α (p<0.001; r=0.57), IL-1β (p<0.05; r=0.44), IL-33 (p<0.001; r=0.65), IL-1Ra (p<0.05; r=0.44), IL-18 (p<0.001; r=0.73) and IL-36α (p<0.001; r=0.68). In contrast, a statistically significant correlation between serum and peritoneal fluid levels in ovarian cancer patients was not found for IL-37 (p>0.05; r=0.32), IL-36β (p>0.05; r=-0.03), IL-36γ (p>0.05; r=0.25), IL-36Ra (p>0.05; r=-0.03) and IL-38 (p>0.05; r=-0.27). Linear regression curves illustrating the aforementioned relationships are shown in **Figure 4**.

### 3.3 Ratio analysis of selected IL-1 members in serum and peritoneal fluid

**Figure 5:**
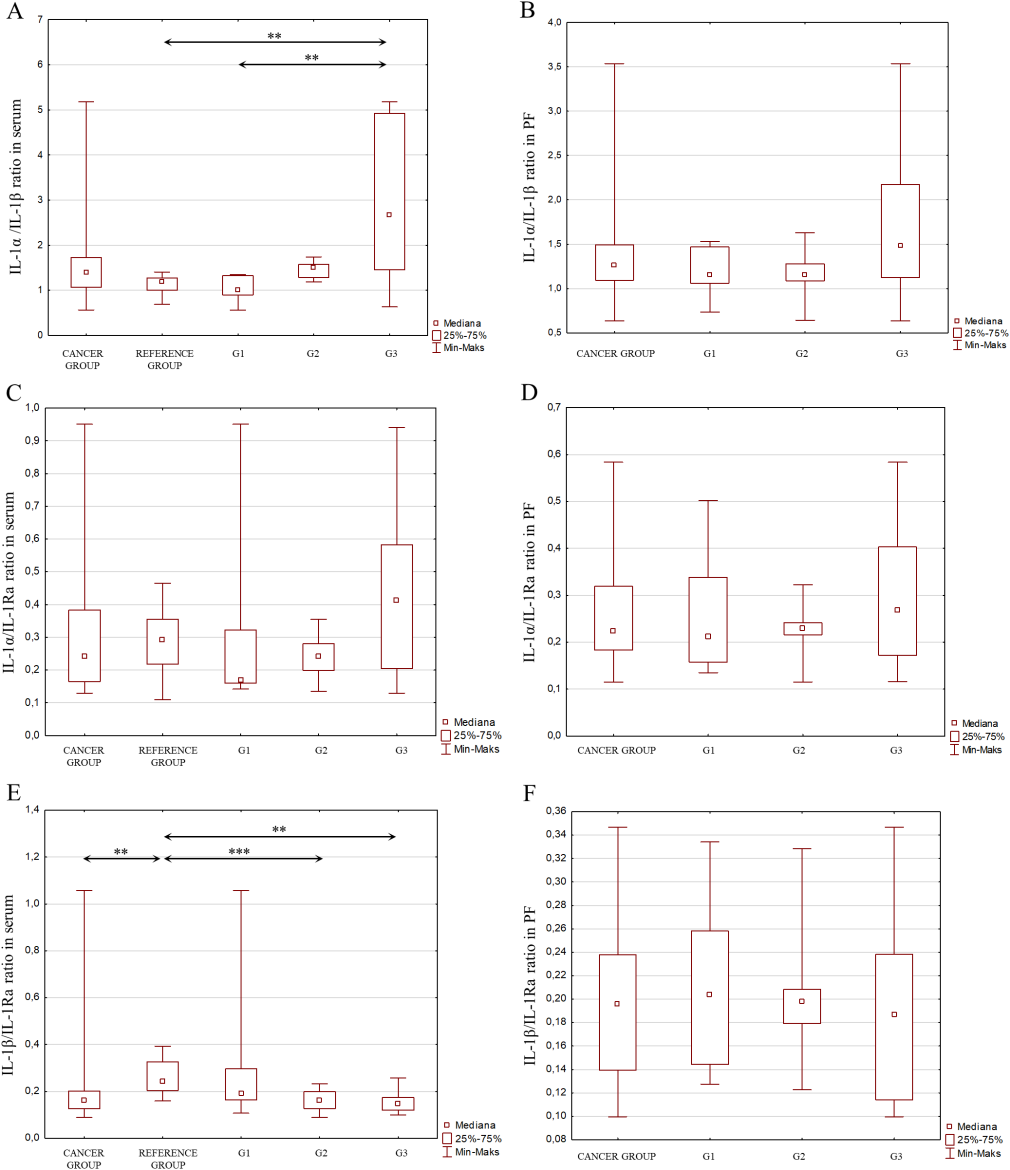
**(A)** IL-1α/IL-1β ratio in the serum from patients in the study and reference groups and the study group depending on the degree of differentiation G1, G2 and G3. **(B)** IL-1α/IL-1β ratio in the peritoneal fluid from patients in the study group depending on the degree of differentiation G1, G2 and G3. **(C)** IL-1α/IL-1Ra ratio in the serum from patients in the study and reference groups and the study group depending on the degree of differentiation G1, G2 and G3. **(D)** IL-1α/IL-1Ra ratio in the peritoneal fluid from patients in the study group depending on the degree of differentiation G1, G2 and G3. **(E)** IL-1b/IL-Ra ratio in the serum from patients in the study and reference groups and the study group depending on the degree of differentiation G1, G2 and G3. **(F)** IL-1β/IL-1Ra ratio in the peritoneal fluid from patients in the study group depending on the degree of differentiation G1, G2 and G3.

**Figure 6:**
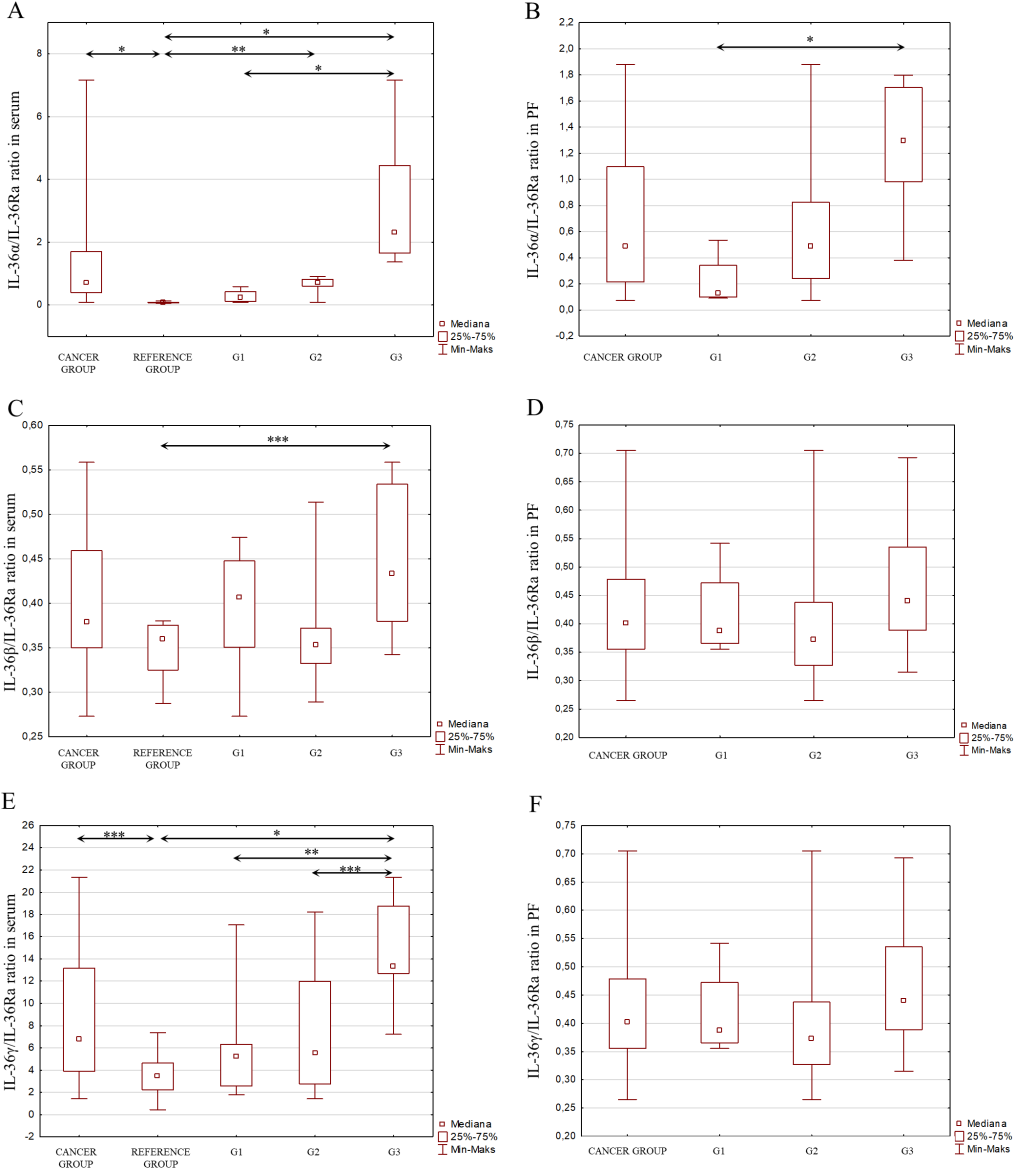
**(A)** IL-36α/IL-36Ra ratio in the serum from patients in the study and reference groups and the study group depending on the degree of differentiation G1, G2 and G3. **(B)** IL-36α/IL-36Ra ratio in the peritoneal fluid from patients in the study group depending on the degree of differentiation G1, G2 and G3. **(C)** IL-36β/IL-36Ra ratio in the serum from patients in the study and reference groups and the study group depending on the degree of differentiation G1, G2 and G3. **(D)** IL-36β/IL-36Ra ratio in the peritoneal fluid from patients in the study group depending on the degree of differentiation G1, G2 and G3. **(E)** IL-36γ/IL-36Ra ratio in the serum from patients in the study and reference groups and the study group depending on the degree of differentiation G1, G2 and G3. **(F)** IL-36γ/IL-36Ra ratio in the peritoneal fluid from patients in the study group depending on the degree of differentiation G1, G2 and G3.

**Figure 7:**
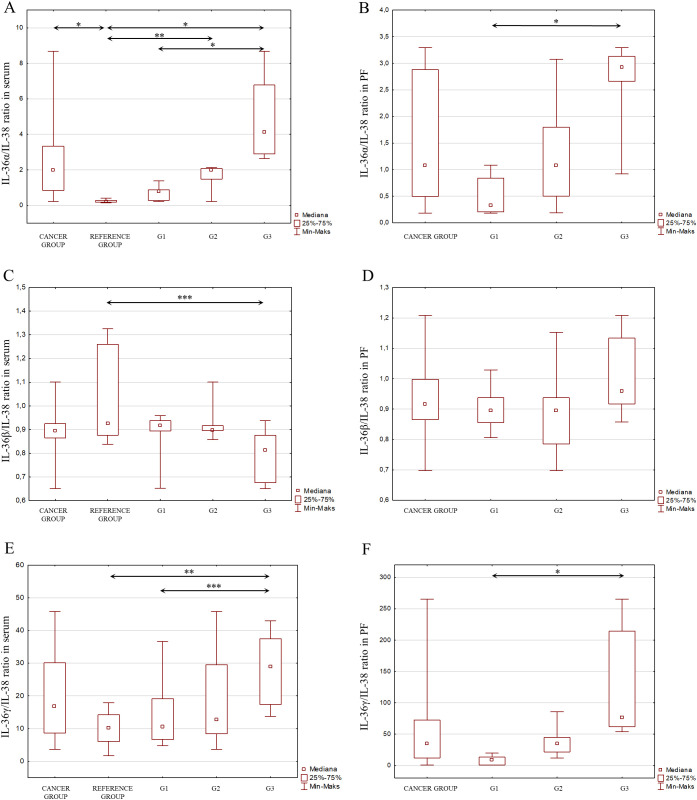
**(A)** IL-36α/IL-38 ratio in the serum from patients in the study and reference groups and the study group depending on the degree of differentiation G1, G2 and G3. **(B)** IL-36α/IL-38 ratio in the peritoneal fluid from patients in the study group depending on the degree of differentiation G1, G2 and G3. **(C)** IL-36β/IL-38 ratio in the serum from patients in the study and reference groups and the study group depending on the degree of differentiation G1, G2 and G3. **(D)** IL-36β/IL-38 ratio in the peritoneal fluid from patients in the study group depending on the degree of differentiation G1, G2 and G3. **(E)** IL-36γ/IL-38 ratio in the serum from patients in the study and reference groups and the study group depending on the degree of differentiation G1, G2 and G3. **(F)** IL-36γ/IL-38 ratio in the peritoneal fluid from patients in the study group depending on the degree of differentiation G1, G2 and G3.

**Figure 8:**
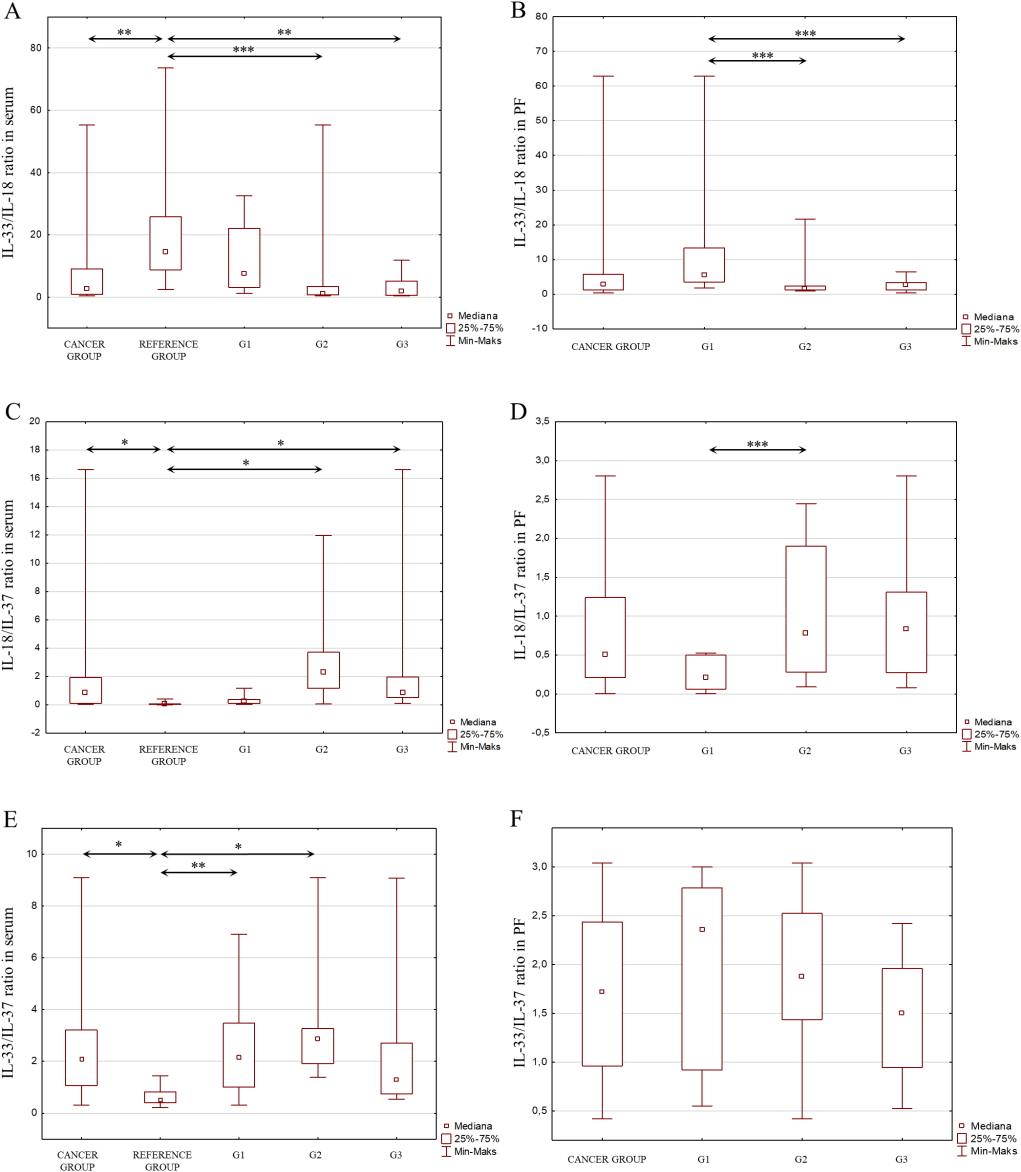
**(A)** IL-33/IL-18 ratio in the serum from patients in the study and reference groups and the study group depending on the degree of differentiation G1, G2 and G3. **(B)** IL-33/IL-18 ratio in the peritoneal fluid from patients in the study group depending on the degree of differentiation G1, G2 and G3. **(C)** IL-18/IL-37 ratio in the serum from patients in the study and reference groups and the study group depending on the degree of differentiation G1, G2 and G3. **(D)** IL-18/IL-37 ratio in the peritoneal fluid from patients in the study group depending on the degree of differentiation G1, G2 and G3. **(E)** IL-33/IL-37 ratio in the serum from patients in the study and reference groups and the study group depending on the degree of differentiation G1, G2 and G3. **(F)** IL-33/IL-37 ratio in the peritoneal fluid from patients in the study group depending on the degree of differentiation G1, G2 and G3.

Considering the signaling pathways of individual IL-1 members in the induction of inflammation and the interactions between these interleukins, as shown in **Figure 2**, an analysis of the IL-1α/IL-1β ratio was also performed, IL-1α/IL-1Ra, IL-1β/IL-1Ra, IL-36α/IL-36Ra, IL-36β/IL-36Ra, IL-36γ/IL-36Ra, IL-36α/IL-38, IL-36β/IL-38, IL-36γ/IL-38, IL-33/IL-18 in both serum and peritoneal fluid. The present analysis showed a statistically significant ratio of the concentrations of individual systems of IL-1 family members between the reference group and the study group in serum and between the different degrees of histological differentiation in both serum and peritoneal fluid. More detailed data obtained from this analysis are shown in **Figures 5**–**8**.

## 4 Discussion

The role of the immune system in the carcinogenesis of many cancers has been the subject of much research for years. It is now known that disorders of the immune system involving immune mediators – interleukins, cytokines, chemokines – are involved in the pathogenesis of many cancers. The role of these mediators is different depending on the organ where the neoplastic transformation has occurred or the clinical stage of the cancer. In the case of ovarian cancer, it has been shown that a chronic inflammatory process involving interleukins, particularly interleukins of the IL-1 family, has an important role in the formation and development of this tumor (29, 31).

Cytokines secreted by type 1 helper T cells (Th1), such as interleukin (IL)-2, IL-12, IL-18, interferon γ (IFN-γ), and tumor necrosis factor (TNF), participate in anti-tumor defense. They participate in the cellular response, influence the activity of NK cells and can lead to the elimination of cancer cells through cytotoxicity. Cytokines secreted by type 2 helper T cells (Th2): IL-4, IL-5, IL-6, IL-10, and IL-13 support the humoral response and participate in immunosuppression by inhibiting the function of NK cells and cytotoxic T cells (Tc) (39). A shift in balance toward Th1 or Th2 can lead to modulation of the immune response and the creation of a microenvironment conducive to cancer cell proliferation and the development of chronic inflammation (40, 41).

*In vitro* studies conducted to date have shown that an imbalance in cytokines secreted by Th1/Th2 lymphocytes affects interactions between SKOV-3 ovarian cancer cells and immune system cells, manifested by changes in the secretion of pro-inflammatory cytokines such as IL-1β, IL-6, and IL-12 (42). Clinical studies conducted by Clendenen TV et al. (43) have shown that many markers of inflammation, including IL-2, IL-4, IL-6, IL-12, and IL-13, may be associated with the risk of ovarian cancer, indicating that inflammation influences the development of this disease.

In this study, the concentrations of all members of the IL-1 family were analyzed for the first time in the serum and peritoneal fluid of women with ovarian cancer (study group) and in the serum of women with benign ovarian tumors (reference group). Patients with ovarian cancer showed significantly higher serum levels of pro-inflammatory interleukins (IL-1α, IL-1β, IL-18, IL-33, IL-36α), anti-inflammatory interleukin (IL-37) and interleukins with antagonistic properties (IL-1Ra, IL-36Ra, IL-38), indicating a regulatory mechanism of these interleukins in the process of ovarian cancer development. In addition, there were statistically significant differences in the concentration of these interleukins in relation to the degrees of histological differentiation. Statistically significantly higher concentrations were observed in patients at stage G3 in relation to stages G1 and G2, which may indicate the influence of the analyzed interleukins in the carcinogenesis of ovarian cancer, especially in advanced stages of the disease. In contrast, statistically significant differences in the concentration of pro-inflammatory interleukins (IL-1α, IL-1β, IL-18, IL-36α, IL-36β, IL-36γ) and an interleukin with antagonistic properties (IL-1Ra) were observed in the peritoneal fluid between G1, G2 and G3 stages of histological differentiation, with the concentration of these interleukins being highest in G3 stage, which may suggest the local involvement of these interleukins in the process of ovarian cancer carcinogenesis. These interleukins seem to have a special role in advanced stages of the disease.

IL-1α in the organism is synthesized by many cell types, especially monocytes, macrophages, fibroblasts and epithelial cells, and for this reason is also found in lower concentrations compared to IL-1β, synthesized especially in the immune response to a pathogen, or also by immune cells, which are components of the tumor microenvironment. IL-1α, unlike IL-1β, was shown to exhibit anti-tumor properties through stimulation and activation of helper T cells, while IL-1β synthesized by ovarian cancer cells promoted increased invasiveness and facilitated the process of formation of secondary metastatic foci under the influence of proangiogenic factors (44). Despite the demonstrated anti-tumor properties of IL-1α, Trabert et al. (45) indicate that persistently elevated IL-1α levels are associated with an increased risk of future ovarian cancer development. In contrast, the authors showed no such correlation for IL-1β and IL-1Ra.

In their study, Santiago et al. (46) assessed the levels of pro-inflammatory cytokines in patients with EOC and healthy women, including IL-1β. The results showed that the concentration of IL-1β is higher in patients with ovarian cancer compared to the concentration of this interleukin in healthy women (p=0.040). The result obtained is consistent with the result of our study. In addition, our results allow us to conclude that higher levels of IL-1β are found in patients with G3 stage of histological differentiation, suggesting a potential pro-tumor function of IL-1β in ovarian cancer. IL-1β has been shown to interact with VEGF via MMPs, thereby promoting angiogenesis. Due to its ability to stimulate angiogenesis, IL-1β has also been shown to contribute to the metastasis of cancer cells, indicating its pro-tumorigenic nature (31, 44).

In addition, Mustea et al. (47) showed significantly higher levels of IL-1Ra in serum and peritoneal fluid in patients with ovarian cancer compared to the control group, which is consistent with our results. The authors indicate that higher IL-1Ra levels are associated with poor prognosis and shortened overall survival. Moreover, based on our study, higher IL-1Ra levels in both serum and peritoneal fluid correlate with the degree of histological differentiation, indicating the involvement of this interleukin in the pathogenesis of ovarian cancer. To the contrary, Woolery et al. (44), however, indicates that IL-1Ra synthesis may be stimulated by IL-1β and/or IL-1α, contributing to its pro-tumor role.

A study on the involvement of IL-37 in the pathogenesis of ovarian cancer was also conducted by Huo et al. (48), who showed elevated levels of IL-37 in patients diagnosed with cancer compared to a healthy group. The concentration of the tested interleukin was associated with the degree of histological differentiation of ovarian cancer and indicated an unfavorable prognosis. In addition, the authors showed that IL-37 correlates with tumor size, lymph node metastasis, positive recurrence and residual tumor size. The authors indicate that IL-37 may be a useful prognostic marker in EOC.

Meanwhile, a study by Orengo et al. (49) on the role of IL-18 in ovarian cancer showed that IL-18 levels are elevated in the serum of patients with epithelial ovarian cancer at the time of diagnosis compared to the levels of this interleukin in the serum of healthy women, indicating a potential role for IL-18 in ovarian cancer carcinogenesis. Moreover, Carbotti et al. (50) demonstrated the presence of IL-18-binding protein in the serum of patients with epithelial ovarian cancer. Their study also showed increased mRNA expression of this protein in epithelial ovarian cancer tumors compared to normal ovarian cells. Similarly, Medina et al. (51) evaluated the expression and cellular origin of IL-18, IL-18 binding protein and IL-18 receptor in physiological ovarian tissues and tumor-lesioned ovarian tissues. The authors demonstrated significantly increased expression of IL-18 and its mRNA in tumor tissue compared to physiological tissue, as well as IL-18 expression in papillary ovarian cancer. The results also showed a significantly higher IL-18/IL-18BP ratio for ovarian cancer tissue and significantly higher IL-18BP expression in physiological ovarian tissue. IL-18 and IL-18BP were expressed in epithelial cells of both tumor and normal ovarian tissues.

Chang et al. (52), in their determination of IL-36α levels by ELISA, conclude that IL-36α in EOC may play a preventive role in the development of cancer, due to the reduced serum levels of this interleukin in EOC patients compared to the control group. The results obtained by Chang et al. are partially consistent with our results. In our study, we confirm that IL-36α levels are present at low levels in patients in the reference group and in patients with ovarian cancer stage G1 and G2, which in these cases may indicate an anti-tumor role for this interleukin. In contrast, in our study, we show that IL-36α levels in patients with stage G3 ovarian cancer are present at high serum levels compared to the reference group, patients with stage G1 and G2 ovarian cancer (where p<0.001), which may indicate a pro-tumor role for IL-36α in this case. On the other hand, Chang et al. (52) analyzed IL-36α concentrations in the serum of patients with EOC – endometrioid ovarian cancer, serous ovarian cancer, mucinous carcinoma, and mixed-type ovarian cancer, whereas in our study we analyzed the concentration of this interleukin in material from patients with serous ovarian cancer, which indicates that the function of IL-36 may vary depending on the type of ovarian cancer, which is an interesting observation. Therefore, IL-36α may exhibit bidirectional functions in ovarian cancer carcinogenesis, both anti-tumor and pro-tumor, and this role may depend on the type of ovarian cancer and the degree of histological differentiation, which requires further in-depth analysis. Considering that IL-36α is a pro-inflammatory interleukin, and chronic inflammation is a well-known factor in carcinogenesis, it therefore appears that with the degree of histological differentiation of ovarian cancer, there is an increase in the inflammatory process, and thus an increase in the concentration of IL-36α, as a mediator of inflammation. On the other hand, the concentration of IL-36β and IL-36γ in the case of the reference group, the group of patients in stage G1, G2 and G3, remained at low levels, not statistically significant between the mentioned groups, which in this case may suggest the lack of involvement of the studied interleukins in the process of carcinogenesis of ovarian cancer, and thus on the anti-tumor role of these interleukins. The present observations confirm the study by Wang et al. (53), who showed that in melanoma and lung cancer, IL-36γ expression inversely correlated with progression in the development of these cancers. The authors showed that IL-36γ exerts anticancer effects by transforming the tumor microenvironment in favor of tumor elimination. An interesting observation concerning IL-36γ is its high concentration in peritoneal fluid, particularly in grade G3 histological differentiation. The result obtained suggests that IL-36γ is not only a passive marker of inflammation, but may also have a potential pro-tumor function in the local inflammatory microenvironment. The direct effect of IL-36γ on tumor microenvironment cells may facilitate tumor proliferation and progression. On the other hand, the association between high IL-36γ concentrations in peritoneal fluid and G3 histological differentiation may be a potential prognostic biomarker indicating advanced cancer. It therefore seems important to confirm the role of IL-36 in tumor progression and to identify the signaling pathways through which it affects cancer cells.

Considering the interactions that occur between cytokines belonging to the IL-1 family, which may affect the direction of their biological action, the stoichiometric concentration relationship of IL-1α/IL-1β, IL-1α/IL-1Ra, IL-1β/IL-1Ra, IL-36α/IL-36Ra, IL-36β/IL-36Ra, IL-36γ/IL-36Ra, IL-36α/IL-38, IL-36β/IL-38, IL-36γ/IL-38, IL-33/IL-18 was evaluated in both serum and peritoneal fluid. The analysis showed significant changes in the concentrations of individual IL-1 family member systems between the reference group and the study group in serum, as well as between different degrees of histological differentiation in both serum and peritoneal fluid, indicating that modulation of IL-1 family cytokine systems may affect the inflammatory process that accompanies the development of ovarian cancer.

The current study indicates an important role for interleukins of the IL-1 family, which are well-known mediators of inflammation in the process of ovarian cancer carcinogenesis, which still remains unclear. Therefore, it seems necessary to conduct further research on the biology of interleukins and their influence on cancer processes.

The present, relatively small sample size is a methodological limitation of this study, which affects its statistical power. A small sample size may prevent the detection of statistically significant differences between the analyzed parameters. Furthermore, the small sample size limits the possibility of generalizing the results to a wider population. Therefore, the conclusions drawn from this study apply only to the study group, and their extrapolation to a wider group of patients requires further research on a larger and fully representative target group.

**Figure 9:**
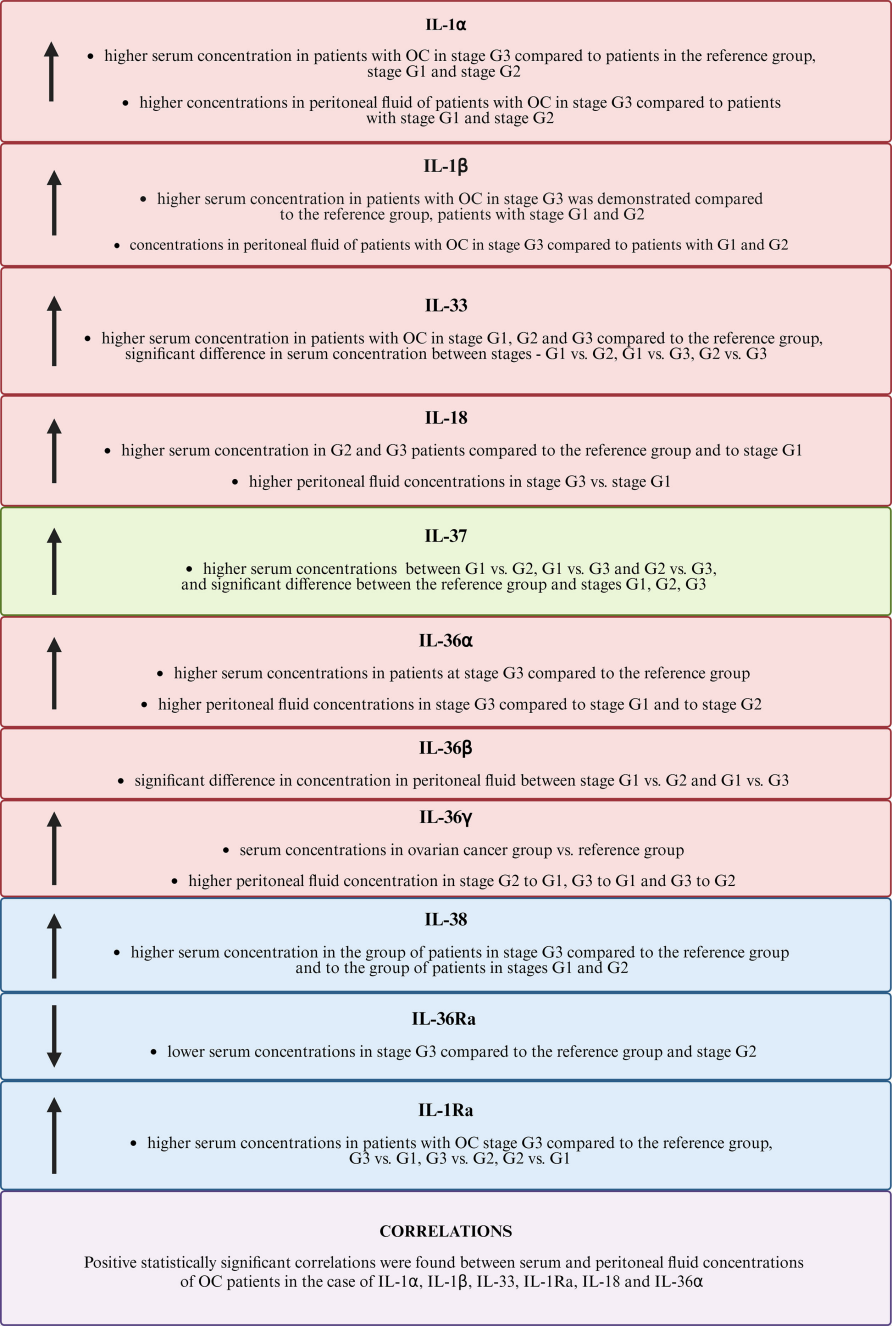
Comparison of the results obtained for the IL-1 family. The figure uses the following arrow symbols: ↑ — indicates an increased concentration of a given IL-1 in the analyzed group, ↓ — indicates a decreased concentration of IL-1 in the analyzed group.

The information obtained from our study provides important information on the important role of individual members of the IL-1 family in the pathogenesis of ovarian cancer (**Figure 9**). Currently, there is a need for further research to clarify the importance of the analyzed parameters in the process of carcinogenesis.

## 5 Conclusion

1. In the interactions taking place between ovarian cancer tumor cells and cells of the immune system, a network of cytokines belonging to the IL-1 family is involved, which indicates the involvement of the studied parameters in the formation and development of ovarian cancer.
2. Disruption of secreted cytokines in serum and peritoneal fluid of ovarian cancer patients may lead to their pro-inflammatory activation, indicating that modulation of cytokines of the IL-1 family may affect the course of the inflammatory process that accompanies the development of cancer.
3. The observed disruptions involving the cytokines studied may represent one of the more important immune mechanisms that promote the development of this cancer, indicating the need for new complex therapies targeting a wide variety of immune response mechanisms, but this requires further, more advanced research.

## Data Availability

The raw data supporting the conclusions of this article will be made available by the authors, without undue reservation.

## References

[1] Sung H Ferlay J Siegel RL Laversanne M Soerjomataram I Jemal A . Global cancer statistics 2020: GLOBOCAN estimates of incidence and mortality worldwide for 36 cancers in 185 countries. CA A Cancer J Clin. (2021) 71:209–49. doi: 10.3322/caac.21660, PMID: 33538338

[2] Bray F Laversanne M Sung H Ferlay J Siegel RL Soerjomataram I . Global cancer statistics 2022: GLOBOCAN estimates of incidence and mortality worldwide for 36 cancers in 185 countries. CA A Cancer J Clin. (2024) 74:229–63. doi: 10.3322/caac.21834, PMID: 38572751

[3] Senent Y Ajona D González-Martín A Pio R Tavira B . The complement system in ovarian cancer: an underexplored old path. Cancers. (2021) 13:3806. doi: 10.3390/cancers13153806, PMID: 34359708 PMC8345190

[4] Smycz−Kubańska M Stępień S Gola J Kruszniewska−Rajs C Wendlocha D Królewska−Daszczyńska P . Analysis of CXCL8 and its receptors CXCR1/CXCR2 at the mRNA level in neoplastic tissue, as well as in serum and peritoneal fluid in patients with ovarian cance. Mol Med Rep. (2022) 26:296. doi: 10.3892/mmr.2022.12812, PMID: 35920183 PMC9435018

[5] Zelisse HS Van Gent MDJM Mom CH De Ridder S Snijders MLH Heeling M . Evaluation of the recently established Dutch nationwide Archipelago of Ovarian Cancer Research biobank. Ann Diagn Pathol. (2025) 74:152411. doi: 10.1016/j.anndiagpath.2024.152411, PMID: 39591762

[6] Tao D Lou S Huang W Sun K Li J Wang Z . Clinical and prognostic significance of FBXL6 expression in ovarian cancer. Gene. (2025) 933:148978. doi: 10.1016/j.gene.2024.148978, PMID: 39368786

[7] Martins Filho A Jammal M Nomelini R Murta E . The immune response in Malignant ovarian neoplasms. Eur J Gynaecol Oncol. (2014) 35:487–91. doi: 10.12892/ejgot24622014 25423690

[8] Charbonneau B Goode EL Kalli KR Knutson KL DeRycke MS . The immune system in the pathogenesis of ovarian cancer. Crit Rev Immunol. (2013) 33:137–64. doi: 10.1615/CritRevImmunol.2013006813, PMID: 23582060 PMC3940260

[9] Baert T Vergote I Coosemans A . Ovarian cancer and the immune system. Gynecol Oncol Rep. (2017) 19:57–8. doi: 10.1016/j.gore.2017.01.002, PMID: 28127584 PMC5247278

[10] Dinca A-L Diaconu A Birla RD Coculescu B-I Dinca V-G Manole G . Systemic inflammation factors as survival prognosis markers in ovarian neoplasm and the relationship with cancer-associated inflammatory mediators—a review. Int J Immunopathol Pharmacol. (2023) 37:3946320231178769. doi: 10.1177/03946320231178769, PMID: 37246293 PMC10226927

[11] Peres LC Townsend MK Birmann BM Conejo-Garcia JR Kim Y Kubzansky LD . Circulating biomarkers of inflammation and ovarian cancer risk in the nurses’ Health studies. Cancer Epidemiol Biomarkers Prev. (2021) 30:710–8. doi: 10.1158/1055-9965.EPI-20-1390, PMID: 33563649 PMC8649851

[12] Aceto GM Cheon D-J . Editorial: Molecular physiopathology of epithelial ovarian cancer: role of inflammation. Front Oncol. (2024) 13:1357842. doi: 10.3389/fonc.2023.1357842, PMID: 38250551 PMC10796465

[13] Liu F Li L Lan M Zou T Kong Z Cai T . Key factor regulating inflammatory microenvironment, metastasis, and resistance in breast cancer: interleukin-1 signaling. Mediators Inflammation. (2021) 2021:1–18. doi: 10.1155/2021/7785890, PMID: 34602858 PMC8486558

[14] Sharma BR Kanneganti T-D . NLRP3 inflammasome in cancer and metabolic diseases. Nat Immunol. (2021) 22:550–9. doi: 10.1038/s41590-021-00886-5, PMID: 33707781 PMC8132572

[15] Kennel KB Bozlar M De Valk AF Greten FR . Cancer-associated fibroblasts in inflammation and antitumor immunity. Clin Cancer Res. (2023) 29:1009–16. doi: 10.1158/1078-0432.CCR-22-1031, PMID: 36399325 PMC10011884

[16] Ehrström A Jansson S Jørgensen MH Wewer V Malham M . The risk of cancer in pediatric-onset immune-mediated inflammatory diseases – A nationwide study. J Autoimmun. (2024) 149:103321. doi: 10.1016/j.jaut.2024.103321, PMID: 39332234

[17] Balescu I Eftimie M Petrea S Diaconu C Gaspar B Pop L . Prognostic significance of preoperative inflammation markers on the long-term outcomes in peritoneal carcinomatosis from ovarian cancer. Cancers. (2024) 16:254. doi: 10.3390/cancers16020254, PMID: 38254745 PMC10814080

[18] Bizzarri N D’Indinosante M Marchetti C Tudisco R Turchiano F Scambia G . The prognostic role of systemic inflammatory markers in apparent early-stage ovarian cancer. Int J Clin Oncol. (2023) 28:314–20. doi: 10.1007/s10147-022-02272-z, PMID: 36417028 PMC9889507

[19] Dinarello CA . Overview of the IL -1 family in innate inflammation and acquired immunity. Immunol Rev. (2018) 281:8–27. doi: 10.1111/imr.12621, PMID: 29247995 PMC5756628

[20] Borthwick LA . The IL-1 cytokine family and its role in inflammation and fibrosis in the lung. Semin Immunopathol. (2016) 38:517–34. doi: 10.1007/s00281-016-0559-z, PMID: 27001429 PMC4896974

[21] Garlanda C Dinarello CA Mantovani A . The interleukin-1 family: back to the future. Immunity. (2013) 39:1003–18. doi: 10.1016/j.immuni.2013.11.010, PMID: 24332029 PMC3933951

[22] Matarazzo L Hernandez Santana YE Walsh PT Fallon PG . The IL-1 cytokine family as custodians of barrier immunity. Cytokine. (2022) 154:155890. doi: 10.1016/j.cyto.2022.155890, PMID: 35462264

[23] Garlanda C Mantovani A . Interleukin-1 in tumor progression, therapy, and prevention. Cancer Cell. (2021) 39:1023–7. doi: 10.1016/j.ccell.2021.04.011, PMID: 33989512

[24] Malik A Kanneganti T . Function and regulation of IL -1α in inflammatory diseases and cancer. Immunol Rev. (2018) 281:124–37. doi: 10.1111/imr.12615, PMID: 29247991 PMC5739076

[25] Hernandez-Santana YE Giannoudaki E Leon G Lucitt MB Walsh PT . Current perspectives on the interleukin-1 family as targets for inflammatory disease. Eur J Immunol. (2019) 49:1306–20. doi: 10.1002/eji.201848056, PMID: 31250428

[26] Zhang H Dhalla NS . The role of pro-inflammatory cytokines in the pathogenesis of cardiovascular disease. IJMS. (2024) 25:1082. doi: 10.3390/ijms25021082, PMID: 38256155 PMC10817020

[27] Yuan X Peng X Li Y Li M . Role of IL-38 and its related cytokines in inflammation. Mediators Inflammation. (2015) 2015:807976. doi: 10.1155/2015/807976, PMID: 25873772 PMC4383490

[28] Martin P Goldstein JD Mermoud L Diaz-Barreiro A Palmer G . IL-1 family antagonists in mouse and human skin inflammation. Front Immunol. (2021) 12:652846. doi: 10.3389/fimmu.2021.652846, PMID: 33796114 PMC8009184

[29] Mantovani A Dinarello CA Molgora M Garlanda C . Interleukin-1 and related cytokines in the regulation of inflammation and immunity. Immunity. (2019) 50:778–95. doi: 10.1016/j.immuni.2019.03.012, PMID: 30995499 PMC7174020

[30] Broderick L Hoffman HM . IL-1 and autoinflammatory disease: biology, pathogenesis and therapeutic targeting. Nat Rev Rheumatol. (2022) 18:448–63. doi: 10.1038/s41584-022-00797-1, PMID: 35729334 PMC9210802

[31] Baker KJ Houston A Brint E . IL-1 family members in cancer; two sides to every story. Front Immunol. (2019) 10:1197. doi: 10.3389/fimmu.2019.01197, PMID: 31231372 PMC6567883

[32] Aggeletopoulou I Tsounis EP Triantos C . Molecular mechanisms underlying IL-33-mediated inflammation in inflammatory bowel disease. IJMS. (2022) 24:623. doi: 10.3390/ijms24010623, PMID: 36614065 PMC9820409

[33] Molofsky AB Savage AK Locksley RM . Interleukin-33 in tissue homeostasis, injury, and inflammation. Immunity. (2015) 42:1005–19. doi: 10.1016/j.immuni.2015.06.006, PMID: 26084021 PMC4471869

[34] Ihim SA Abubakar SD Zian Z Sasaki T Saffarioun M Maleknia S . Interleukin-18 cytokine in immunity, inflammation, and autoimmunity: Biological role in induction, regulation, and treatment. Front Immunol. (2022) 13:919973. doi: 10.3389/fimmu.2022.919973, PMID: 36032110 PMC9410767

[35] Fang Z Jiang J Zheng X . Interleukin-1 receptor antagonist: An alternative therapy for cancer treatment. Life Sci. (2023) 335:122276. doi: 10.1016/j.lfs.2023.122276, PMID: 37977354

[36] Silvério D Gonçalves R Appelberg R Saraiva M . Advances on the role and applications of interleukin-1 in tuberculosis. mBio. (2021) 12:e03134–21. doi: 10.1128/mBio.03134-21, PMID: 34809460 PMC8609357

[37] Gurung P Kanneganti T-D . Autoinflammatory skin disorders: the inflammasome in focus. Trends Mol Med. (2016) 22:545–64. doi: 10.1016/j.molmed.2016.05.003, PMID: 27267764 PMC4925313

[38] Mantovani A Barajon I Garlanda C . IL -1 and IL -1 regulatory pathways in cancer progression and therapy. Immunol Rev. (2018) 281:57–61. doi: 10.1111/imr.12614, PMID: 29247996 PMC5922413

[39] Zhang Y Zhang Y Gu W He L Sun B . Th1/th2 cell’s function in immune system. In: Sun B , editor. T Helper Cell Differentiation and Their Function. Advances in Experimental Medicine and Biology. Springer Netherlands, Dordrecht (2014). p. 45–65. doi: 10.1007/978-94-017-9487-9_3, PMID: 25261204

[40] Striz I Brabcova E Kolesar L Sekerkova A . Cytokine networking of innate immunity cells: a potential target of therapy. Clin Sci. (2014) 126:593–612. doi: 10.1042/CS20130497, PMID: 24450743

[41] Cosmi L Maggi L Santarlasci V Liotta F Annunziato F . T helper cells plasticity in inflammation. Cytometry Pt A. (2014) 85:36–42. doi: 10.1002/cyto.a.22348, PMID: 24009159

[42] Mielczarek-Palacz A Sikora J Kondera-Anasz Z Mickiewicz P Mickiewicz A . Effect of Th1/Th2 cytokine administration on proinflammatory SKOV-3 cell activation. aoms. (2016) 6:1337–47. doi: 10.5114/aoms.2015.53143, PMID: 27904527 PMC5108376

[43] Clendenen TV Lundin E Zeleniuch-Jacquotte A Koenig KL Berrino F Lukanova A . Circulating inflammation markers and risk of epithelial ovarian cancer. Cancer Epidemiol Biomarkers Prev. (2011) 20:799–810. doi: 10.1158/1055-9965.EPI-10-1180, PMID: 21467242 PMC3089656

[44] Woolery KT Kruk PA . Ovarian epithelial-stromal interactions: role of interleukins 1 and 6. Obstetr Gynecol Int. (2011) 2011:1–9. doi: 10.1155/2011/358493, PMID: 21765834 PMC3135012

[45] Trabert B Pinto L Hartge P Kemp T Black A Sherman ME . Pre-diagnostic serum levels of inflammation markers and risk of ovarian cancer in the Prostate, Lung, Colorectal and Ovarian Cancer (PLCO) Screening Trial. Gynecol Oncol. (2014) 135:297–304. doi: 10.1016/j.ygyno.2014.08.025, PMID: 25158036 PMC4254357

[46] Santiago AE Paula SOCD Carvalho ATD Cândido EB Furtado RDS Silva Filho ALD . Systemic inflammatory patterns in ovarian cancer patients: analysis of cytokines, chemokines, and microparticles. Rev Bras Ginecol Obstet. (2023) 45:e780–9. doi: 10.1055/s-0043-1772590, PMID: 38141599 PMC10748511

[47] Mustea A Pirvulescu C Könsgen D Braicu EI Yuan S Sun P . Decreased IL-1 RA concentration in ascites is associated with a significant improvement in overall survival in ovarian cancer. Cytokine. (2008) 42:77–84. doi: 10.1016/j.cyto.2008.01.011, PMID: 18329282

[48] Huo J Hu J Liu G Cui Y Ju Y . Elevated serum interleukin-37 level is a predictive biomarker of poor prognosis in epithelial ovarian cancer patients. Arch Gynecol Obstet. (2017) 295:459–65. doi: 10.1007/s00404-016-4258-8, PMID: 27975129

[49] Orengo AM Fabbi M Miglietta L Andreani C Bruzzone M Puppo A . Interleukin (IL)-18, a biomarker of human ovarian carcinoma, is predominantly released as biologically inactive precursor. Intl J Cancer. (2011) 129:1116–25. doi: 10.1002/ijc.25757, PMID: 21710494

[50] Carbotti G Barisione G Orengo AM Brizzolara A Airoldi I Bagnoli M . The IL-18 antagonist IL-18–binding protein is produced in the human ovarian cancer microenvironment. Clin Cancer Res. (2013) 19:4611–20. doi: 10.1158/1078-0432.CCR-13-0568, PMID: 23873689

[51] Medina L Rabinovich A Piura B Dyomin V Shaco Levy R Huleihel M . Expression of IL-18, IL-18 binding protein, and IL-18 receptor by normal and cancerous human ovarian tissues: possible implication of IL-18 in the pathogenesis of ovarian carcinoma. Mediators Inflammation. (2014) 2014:1–8. doi: 10.1155/2014/914954, PMID: 24963217 PMC4052106

[52] Chang L Guo R Yuan Z . IL-36α suppresses proliferation of ovarian cancer cells. Tumour Biol. (2017) 39:101042831770691. doi: 10.1177/1010428317706918, PMID: 28621240

[53] Wang X Zhao X Feng C Weinstein A Xia R Wen W . IL-36γ Transforms the tumor microenvironment and promotes type 1 lymphocyte-mediated antitumor immune responses. Cancer Cell. (2015) 28:296–306. doi: 10.1016/j.ccell.2015.07.014, PMID: 26321222 PMC4573903

[54] Su Z Tao X . Current understanding of IL-37 in human health and disease. Front Immunol. (2021) 12:696605. doi: 10.3389/fimmu.2021.696605, PMID: 34248996 PMC8267878

